# Neuropsychobiology of fear-induced bradycardia in humans: progress and pitfalls

**DOI:** 10.1038/s41380-024-02600-x

**Published:** 2024-06-11

**Authors:** Simone Battaglia, Claudio Nazzi, Tina B. Lonsdorf, Julian F. Thayer

**Affiliations:** 1 Center for Studies and Research in Cognitive Neuroscience, Department of Psychology, University of Bologna, Bologna, Italy; 2 Department of Psychology, University of Torino, Torino, Italy; 3 Department of Systems Neuroscience, University Medical Center Hamburg Eppendorf, Hamburg, Germany; 4 Department of Psychology, Section for Biological Psychology and Cognitive Neuroscience, University of Bielefeld, Bielefeld, Germany; 5 Department of Psychological Science, 4201 Social and Behavioral Sciences Gateway, University of California, Irvine, CA USA; 6 Department of Psychology, The Ohio State University, Columbus, OH USA

**Keywords:** Neuroscience, Psychology, Physiology

## Abstract

In the last century, the paradigm of fear conditioning has greatly evolved in a variety of scientific fields. The techniques, protocols, and analysis methods now most used have undergone a progressive development, theoretical and technological, improving the quality of scientific productions. Fear-induced bradycardia is among these techniques and represents the temporary deceleration of heart beats in response to negative outcomes. However, it has often been used as a secondary measure to assess defensive responding to threat, along other more popular techniques. In this review, we aim at paving the road for its employment as an additional tool in fear conditioning experiments in humans. After an overview of the studies carried out throughout the last century, we describe more recent evidence up to the most contemporary research insights. Lastly, we provide some guidelines concerning the best practices to adopt in human fear conditioning studies which aim to investigate fear-induced bradycardia.

## Introduction

**Fig. 1:**
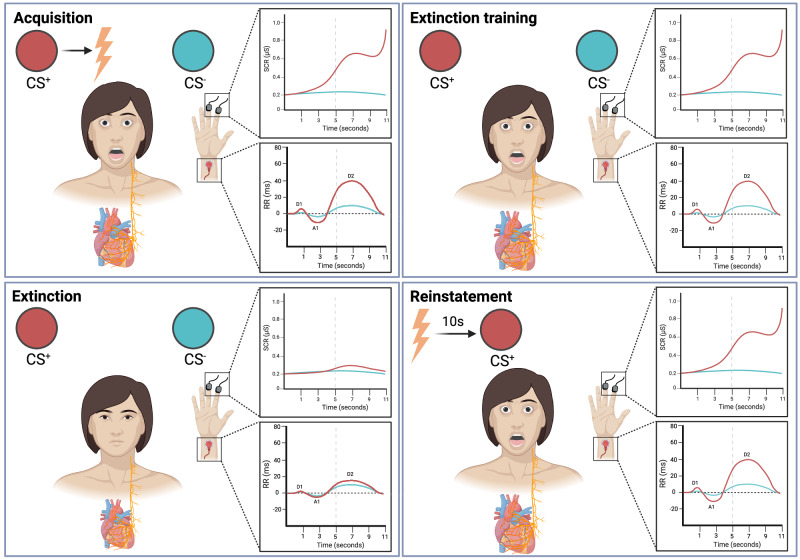
Psychophysiological conditioned responses. Fear acquisition is achieved by presenting a neutral stimulus (conditioned stimulus, CS+) with a negative consequence, like a shock to the wrist (unconditioned stimulus, US). This causes fear learning to take place, which manifests the development of conditioned responses to the conditioned stimulus (CS+), such as increased skin conductance response and fear-induced bradycardia, even in the absence of a threatening outcome - such as during extinction. During extinction training, however, repeated presentations of CSs without painful sensations bring physiological responses back to baseline levels. Administering the US after the extinction phase, however, triggers a reinstatement effect, that is, the CS+ typically elicits physiological activation again. The figure was created using BioRender.com.

Fear is an invaluable resource that protects living beings from dangerous situations. It is not only relevant for innately fear-inducing stimuli, but it is also useful in rapidly creating associations between neutral stimuli and unpleasant outcomes, thus allowing organisms to adapt to an ever-changing environment [1, 2]. This process is called fear learning, and it is crucial to predict aversive events and ensure survival [3–5]. To study physiological changes associated with fear learning in both animals and humans, researchers of several disciplines use fear conditioning, an experimental paradigm that allows investigation of these changes in a controlled environment [6]. The first instance of human fear conditioning can be found in the famous, albeit very cruel, ‘little Albert’ experiment, in which a child was conditioned to fear a rat by pairing its presence with a loud, startling noise [7]. This experimental procedure derived from the work of Ivan Pavlov, who studied the processes underlying appetitive conditioning in animals [8]. Fear conditioning may occur when a previously neutral conditioned stimulus (CS) is paired with an intrinsically unpleasant or even harmful stimulus referred to as unconditioned stimulus (US), which elicits unconditioned responses (UR). After this association, the CS comes to elicit conditioned responses (CR), which are similar to the UR [9, 10]. As of today, most fear conditioning studies in humans use not one but two CSs, commonly termed CS+, associated with the US, and CS−, as a control stimulus but never paired with the US. Depending on the number of times that the CS+ is paired with the US, the paradigm consists of continuous pairings (when the CS+ is always paired with the US) or a partial reinforcement schedule (when the CS+ is reinforced only on some trials) [11]. Moreover, depending on the timing of US delivery, it is possible to distinguish between delay conditioning – when the US is delivered near the end of the CS+ – and trace conditioning – when the US is delivered after the CS+ offset [12, 13]. Additionally, fear conditioning paradigms can be defined as uninstructed, when participants begin the experiment without knowing which of the two stimuli will be paired with the US, or instructed, meaning that prior to the experiment participants will be taught which CS will be paired with the US [12]. During extinction training, the CSs are presented, without the US. This engenders a new inhibitory learning of the CS-US association, so that the CS+ will no longer elicit CRs [14, 15]. Finally, retention tests allow to investigate the presence or absence of CRs after fear acquisition or extinction training [12, 16]. These procedures constitute an important experimental paradigm for the behavioral and cognitive sciences (Fig. 1). Thanks to decades of scientific research conducted by using the fear conditioning paradigm in humans, remarkably vast and deep understanding of fear itself and its related processes, like learning, memorization, retrieval, extinction, and reconsolidation has come to life [12, 17].

In human fear conditioning research, psychophysiological indices are extensively used thanks to their advantage of not being subject to self-report biases [12, 18], since implicit and explicit learning rely on different neural pathways [19]. The most commonly used index of fear conditioning is the electrodermal activity, usually measured as skin conductance response (SCR), which is a phasic response to a stimulus and reflects enhanced autonomic arousal [20]. In fear conditioning paradigms, CS+ presentations typically elicit greater responses than CS- presentations. Among psychophysiological indices, fear potentiated startle (FPS) [21–23] is another valuable measure that is used to quantify fear learning. The FPS reflex is elicited by the administration of a sudden and startling event (e.g., sound or air puff) and is measured with electromyography (EMG) of the orbicularis oculi muscle, which is responsible for eyelid closure. The term FPS refers to the fact that the startle reflex is larger when administered during the presentation of a threatening stimulus as compared to administration during presentation of a neutral or pleasant stimulus [24]. Another psychophysiological measure is the pupillary response [25]. Pupil diameter is primarily influenced by optical reflexes for light and distance, but it is also associated with mental processes like emotional learning [26]. In the context of fear conditioning, exposure to a threatening stimulus engenders stronger pupil dilation compared to neutral stimuli [27, 28]. As opposed to SCR, which is modulated slowly, pupillary responses are fast and reflect a measure of psychological arousal [29, 30]. This approach is ideal in the case of paradigms with short intervals between stimuli or when assessing overlapping responses arising from subsequent stimuli [12]. Moreover, since pupillary responses can be measured by eye-tracking or pupillometry, they can easily be employed even in magnetic resonance imaging studies [12]. Finally, even though heart rate (HR) is a physiological signal that has been known for centuries, its application in quantifying fear conditioning in humans is still in its early stages. In the last decade, HR has been employed more frequently as a psychophysiological measure to assess human fear conditioning, however with mixed results that in part depend on the analysis methods and paradigms used (for an example, see Castegnetti et al. [31]).

With this review, we aim at exploring the major advancements in the field of fear-induced bradycardia in the last century by focusing on the methodological improvements of the last twenty years, highlighting new cutting-edge approaches. Specifically, we will (i) examine studies that investigated the role of fear-induced bradycardia in fear conditioning experiments, (ii) interpret the results that were obtained by the different types of methodologies applied, (iii) extract common physiological patterns among these studies to draw the current state of the art of the phenomenon, and (iv) lastly, we aim to provide specific guidelines, especially regarding the methodologies used to analyze heart rate in fear conditioning work. Finally, the following sections of this review will provide a description of the most theoretically and methodologically influential studies, which served to develop theories and techniques still used today.

### Cardiac neuroanatomy

Classical theories state that the sympathetic and parasympathetic branches of the autonomic nervous system act independently, however more recent evidence suggests that heart dynamics are regulated by different reflex pathways that combine efferent and afferent information [32, 33]. This cardiac system is divided in three major constituents: the central nervous system, intrathoracic extracardiac neuronal pools, and neurons located directly on the heart, also defined as the intrinsic cardiac nervous system. As its definition suggests, it can act independently from higher-order structures [34]. The function of this system depends on both afferent and efferent neurons that, alongside local circuit neurons are found on various sites throughout the network, interact between each other to ensure appropriate heart function [32].

The first set of these neurons are afferent or sensory neurons, which relay information to higher order structures about mechanical and chemical changes of the heart [35]. They present different characteristics depending on the location of their neurites and their soma. For example, atrial and ventricular neurons that send signals to the nodose ganglion of the vagus nerve transmit mechanical information [36]. On the other hand, neurons located in dorsal root ganglia with projections to the four chambers of the heart display higher frequency activity compared to nodose ganglia ones [37], and display more activation when transmitting information [38]. Alongside these, neurons in intrathoracic ganglia communicate aortic wall dynamics [39–42]. Likewise, unipolar neurons in ganglionated plexi on the heart relay sensory information, even though these type of neurons represent only 10% of the total population contained in the heart [43].

Efferent or motor neurons, on the other hand, provide control over heart muscle fibres and coronary vessels [44]. More precisely, sympathetic neurons originate from the reticular formation in the brainstem and project to postganglionic neurons [45]. These latter neurons display an ability to regulate heart dynamics even when disconnected from higher-order structures [46]. Moreover, they control the entirety of the heart [47]. Parasympathetic neurons originate instead from the nucleus ambiguus and the dorsal motor nucleus [38, 48], working alongside postganglionic neurons located in ganglionated plexi [43, 49]. Similarly to their sympathetic counterpart, they provide diffuse control over the various regions of the heart [50, 51]. This redundancy, true to both types of autonomic regulation neurons, ensures that appropriate modulation can happen even in the face of focal lesions [32].

Local circuit neurons are a set of neurons that is not dedicated to the relay of sensory or motor information, but rather to the integration of signals from afferent and efferent neurons [52]. These are found in intrathoracic ganglia and in ganglionated plexi on the heart and communicate between each other constantly [52, 53].

On a larger scale, the autonomic nervous system interacts dynamically with the central nervous system in order to promote control of the heart, as hypothesized by the neurovisceral integration model [54, 55]. In the case of threatening situations, a complex circuitry determining the interplay between the central and autonomic nervous system gets involved. Specifically, sympathoexcitatory neural circuits undergo disinhibition, which results in fear responses, and prefrontal cortex and the amygdala engender excitatory control of the parasympathetic downward regulation. After threat detection, these structures regulate heart rate through a pathway that involves the nucleus ambiguus, the dorsal nucleus of the vagus nerve, and the vagus nerve itself, whose endpoint is located on the sinoatrial node of the heart, thus eliciting fear induced bradycardia [56]. On the other hand, in the presence of neutral stimuli, the prefrontal cortex identifies safety cues and exercises inhibition over sympathoexcitatory subcortical networks by means of vagal control [56]. This circuit highlights the intertwined relationship between central and peripheral nervous systems, which supports autonomic regulation in the brain-heart axis and underlines the influence of high level cognitive processes on hear rate [57].

In conclusion, this tightly interconnected system allows for a constant and precise monitoring and regulation of heart rate. Nonetheless, this stability may be influenced when the organism is exposed to threatening stimuli, giving rise to fear induced bradycardia.

### The history of fear induced bradycardia

The first reported evidence of a cardiac conditioned response dates back to 1900, when Sherrington [58] incidentally observed a reduction in heart rate in dogs when they were presented with the sound of an inductorium that was previously used to apply shocks. Since then, a wide range of techniques and experimental protocols have been used to study this physiological phenomenon to investigate its underpinnings. In humans, the seminal study by Notterman et al. [59] reported conditioned cardiac responses in different moments of a fear conditioning paradigm. The authors discussed how they created a CS by combining a neutral tone with a shock to the hand, while recording heart rate. Their results highlighted a decrease in heart rate between the pre-tone period and the post-tone period, laying the foundations for the study of what we currently describe as ‘fear induced bradycardia’. It should be noted that heart rate data for this study was collected by measuring the distance between consecutive heartbeats in centimeters, and the shock used as US lasted for six seconds. After almost seventy years, researchers have come a long way in terms of experimental tools, techniques, and ethics [12, 60]. These early studies provide a solid basis for the study of fear induced bradycardia (see Table 1 for a summary), even though the methods used are less sophisticated than those available today.

**Table 1:** A summary of findings on fear-induced bradycardia in studies from the past century.

Study	Participants (N)	Fear conditioning paradigm	Psychophysiological Measures of fear	CS	US	Analysis method	Main findings
Headrick and Graham [70]	60	Partial reinforcement delay conditioning	HR	Tones	Shock	BPM	Bradycardia for CS+ in the Fast Breathing group
Obrist et al*.* [66]	26	Delay conditioning	HR, respiration, chin EMG activity	Light	Shock	BPM	Bradycardia for CS
Furedy and Poulos [64]	24	Partial reinforcement delay conditioning	HR	Tones	Body tilt	Heart rate/second	Bradycardia for CS+
Hugdahl [72]	40	Delay conditioning	HR	Visual stimuli (fear-relevant: spider/snake; fear-irrelevant: geometrical shapes)	Shock or threat of shock	BPM	Bradycardia for CS+ in the fear-relevant shock group
Frederickson and Ohman, 1979	32	Delay conditioning	HR, SCR	Visual stimuli (fear-relevant: spider/snake; fear-irrelevant: flower/mushroom)	Shock	BPM	Greater acceletarion for phobic CSs+ compared to non-phobic CSs+
Klorman and Ryan [73]	24	Delay conditioning	HR	Tones	Picture of mutilated body	Heart rate/second	Bradycardia for CS+ in participants with high fear of mutilation
Hodes et al. [75]	148	Delay conditioning	HR, SCR	Visual stimuli (fear relevant or neutral)	Tones + vibratory stimulus to finger	BPM	Three patterns of cardiac activity: Accelerators, Decelerators and Moderate Decelerators
Dimberg*,* [67]	37	Delay conditioning	HR, SCR, currogator end zygomatic responses	Visual stimuli (angry and happy facial expressions)	Tone	BPM	Tachycardia for CS+ during extinction for angry faces only
Sandin and Chorot [68]	79	Delay conditioning	HR	Visual stimuli (spiders and snakes)	Tones (115 dB or 95 dB)	BPM	Tachycardia for CS+ but only when the US is set at 115 dB
Hamm et al*.* [65]	60	Delay conditioning	HR, SCR, eyeblink responses, corrugator responses	Visual stimuli (IAPS)	Shock	BPM	Bradycardia for CS+ in the first trials of acquisition, then tachycardia
Stegen et al*.* [69]	56 (females only)	Delay conditioning	HR, respiration	Fear relevant or neutral imagery scripts	CO_2_ enriched air	BPM	No effects on HR

The knowledge needed for the development of refined experimental and methodological protocols comes from a small number of key studies from the 50s to the end of the century. In particular, Notterman and colleagues were one of the first research groups to investigate fear induced bradycardia with specific experimental research designs. Authors followed up their first study with additional evidence and suggested that a conditioned cardiac response can also be observed under partial reinforcement schedules [61]. The idea of a strong conditioned cardiac response was further supported by the evidence that this is independent of the CS-US interval. [62]. Finally, it was shown that the extinction of fear-induced bradycardia is much more rapid following instructed extinction, as after only 5 trials fear conditioned bradycardia has disappeared, while following uninstructed extinction it persists even after 10 trials [63].

Some subsequent studies found the same pattern of conditioned cardiac deceleration [64–66], while others found the opposite or no modulation of cardiac activity [67–69]. This heterogeneity in results has been resolved thanks to recent studies. For instance, different research groups found a common link between fear-induced bradycardia and higher arousal. As an example of this connection, when trained to control their breathing, only participants that are asked to breathe at a faster pace (46 cycles per minute) – simulating a higher arousal condition – than regular breathing (14 cycles per minute) show fear-induced bradycardia [70]. Moreover, the pictures used as CSs play a big factor in the generation of fear-induced bradycardia, which could be observed only in groups that were exposed to fear-relevant pictures (i.e., snakes and spiders) or objects of specific fears or phobias as opposed to neutral imagery [71–73]. However, when conditioned, even neutral pictures can elicit fear-induced bradycardia [31, 74].

To conclude, it is possible to draw a line that connects all these historical studies under a single light of evidence: the cardiac deceleration was found only in the groups in which participants underwent more arousing conditions, such as fear-relevant stimuli and conditioned stimuli. Moreover, Hodes, Cook, and Lang [75] were able to differentiate between accelerators, decelerators, and moderate decelerators by means of a cluster analysis on 148 participants. Participants were defined as ‘accelerators’ if their heart rate was faster after exposure to the CS+ compared to the CS-, and ‘decelerators’ if their heart rate was slower. Meanwhile, ‘moderate decelerators’ showed a moderate heart rate slowing. Furthermore, acceleration was defined as a defensive response, preparing the individual for motor activity, and deceleration as an orienting response, predisposing for sensory intake [75]. Based on all the classical evidence illustrated above it was possible to pave the way for current studies on the phenomenon of fear induced bradycardia. Indeed, researchers in this field have come to more consistent results thanks to the preliminary data coming from these classical studies [60].

Currently, a series of studies have demonstrated that fear conditioned stimuli are related to heart rate deceleration. However, there are still a few studies that suggest the opposite, which will be discussed in detail in the next paragraphs.

## Settling the dispute: heart rate deceleration following CS presentation

### Beats per minute change

The most classical and widely used method to record cardiac rhythm variations is the assessment of beats per minute changes, which is measured by converting inter-beat-intervals into beats per minute, weighting each interval proportionally to the amount of time it occupies [76]. The following studies have provided early evidence towards fear conditioned bradycardia assessed by this analysis method (see Table 2 for a summary).

**Table 2:** A summary of findings on fear-induced bradycardia in studies that use beats per minute change to assess heart rate variations.

Study	Participants (N)	Fear Conditioning Paradigm	Psychophysiological Measures of Fear	CS	US	Analysis method	Main findings
Wamsley and Antrobus [77]	43	Delay and trace conditioning	HR	Tones	Car horn	BPM	Bradycardia for CS+ in delay conditioning, tachycardia for CS+ in trace conditioning
Costa et al. [78]	71	Threat reversal	HR, SCR, FPS, corrugator responses	Visual stimuli (geometrical shapes)	Threat of shock	BPM	Bradycardia for original CS+ and for new CS+ after reversal
Szeska et al. [84]	80	Partial reinforcement delay conditioning	HR, FPS	Visual stimuli (geometrical shapes)	Loud tone	BPM	Bradycardia for CS+ in the fear learning group

One of the first evidence in fear conditioning framework was provided by Wamsley and Antrobus [77] which aimed at investigating the characteristics of memory reactivation in sleeping humans. To do so, they recruited a total of 43 participants which were split in two groups: a delay conditioning group and a trace conditioning group. The results show that trace conditioned participants showed an increased heartbeat for the CS+ compared to the CS−, while delay conditioned participants showed a decrease in heart beats per minute for the CS+ compared to the CS−. Therefore, we can conclude that by using a delay fear conditioning paradigm a decreased heartbeat during CS+ presentation could be observed. This is an important finding, as most of the following studies employ delay conditioning paradigms as well.

To study instructed fear conditioning and reversal of CS associations, Costa et al. [78] recruited 71 participants to take part in a fear conditioning experiment where no US was delivered but participants were warned that they could be exposed to a shock when viewing two out of four stimuli (based on either color or shape). Immediately after this phase, instructions were changed, such that the other parameter (i.e., color or shape) was now predictive of receiving a shock. During the first phase, a greater initial deceleration for CSs+ was observed. A change in instructions resulted in greater deceleration for the new CSs+ while new CSs- now promoted acceleration. This study established how verbal instructions are effective in eliciting and modulating fear conditioned bradycardia.

Fear-induced bradycardia emerges in response to the detection of a threatening stimulus, as part of a generalized fear state of freezing [79, 80]. As animal research highlights a link between freezing and both fear-induced bradycardia [81] and increased FPS responses [82, 83], Szeska et al. [84] suggested this may be true for humans as well. To test this hypothesis, they had participants undergo a fear conditioning paradigm, and additionally administered transcranial vagus nerve stimulation (tVNS) during the extinction phase. tVNS is a non-invasive brain stimulation technique that involves the application of electrical currents through surface electrodes. It has been shown that it influences various regions of the brain involved in anxiety and mood regulation [85] and facilitates the reduction of defensive responses during extinction compared to sham stimulation [86–89]. The results show how fear-induced bradycardia is associated with potentiation of the FPS reflex, suggesting the emergence of what the authors call “attentive immobility”, a defense strategy that can be observed in animals when danger is unavoidable [90, 91]. Moreover, fear-induced bradycardia was observed in the fear learning group only, which was exposed to the CS+ immediately followed by the US, but not in the control group, in which the CS+ and the US were temporally separated by an interval, and deceleration was strongest just before US onset. The tVNS significantly impacted on extinction, as the tVNS fear learning group showed a faster attenuation of cardiac deceleration compared to the sham fear learning group. In conclusion, the results from this study suggest that fear-induced bradycardia is specific to fear learning, and that it might be mediated by the parasympathetic pathway.

All in all, data collected from these studies reflects the idea of fear-induced bradycardia as viable evidence in the assessment of conditioned responses. It appears to be useful not just in strictly classical fear conditioning studies, but also in sleep studies [77] and in instructed conditioning studies [78]. Furthermore, tVNS revealed the role of the parasympathetic pathway in the regulation of fear-induced bradycardia [84].

### Accelerators and decelerators

A series of studies using cluster analysis have shown that participants could be separated in two different sub-groups, namely accelerators and decelerators, depending on their cardiac activity in response to conditioned stimuli [75, 92–95] (see Table 3 for further details). More specifically, participants classified as ‘accelerators’ show a faster heart rate when exposed to threatening stimuli, while the opposite is true for ‘decelerators’. A first example comes from a study by Moratti and Keil [94] who attempted to discriminate between brain activations associated with cardiac accelerators and decelerators. A cluster analysis allowed to separate participants in an accelerators and a decelerators group, based on the cardiac pattern shown during CS+ presentations in acquisition training. Accelerators showed a greater acceleration to the CS+ compared to the CS- during fear acquisition training, and a deceleration to the CS+ during extinction training. On the other hand, decelerators showed no significant difference between CS+ and CS- during neither fear acquisition training nor extinction training. Based on these data, it was suggested that only accelerators show a modulation of cardiac activity. Notwithstanding, decelerators showed greater steady state visual evoked field amplitude in the frontal region during CS+ presentation and just before US administration. On the other hand, accelerators did not show a differential response during fear acquisition, but manifested an increase in steady state visual evoked field amplitude in the left parietal region in response to the CS+ during extinction. It should, however, be noted that this study has some limitations. First and foremost, only 3 out of 17 participants were aware of the CS+/US contingency at the end of the experiment. Secondly, HR to the CS+ in accelerators did not go over the baseline level, it simply differed from the CS- response which prompted a greater deceleration. Lastly, the cluster analysis used to differentiate between accelerators and decelerators may have merely separated participants that showed successful conditioning from those who did not. These strong limitations of the study, however, prevent from drawing a straightforward conclusion.

**Table 3:** A summary of findings on fear-induced bradycardia in studies that identify accelerators and decelerators.

Study	Participants (N)	Fear Conditioning Paradigm	Psychophysiological Measures of Fear	CS	US	Analysis Method	Main Findings
Moratti and Keil [94]	17	Delay conditioning	HR	Visual stimuli (gratings)	Tone	BPM	Bradycardia for CS+ in accelerators during extinction
Moratti et al. [93]	20	Delay conditioning	HR	Visual stimuli (gratings)	Tone	BPM	Bradycardia for CS+ in decelerators, tachycardia for accelerators
Lopez et al. [92]	73	Delay conditioning	HR, SCR, FPS	Visual stimuli (erotic or threatening)	Shock pulse	BPM	Bradycardia for CS+ in accelerators
Sevenster et al. [99]	39	Partial reinforcement delay conditioning	HRV, SCR, FPS	Visual stimuli (spiders)	Shock pulse	BPM for each 0.5 of the 7s following the CS minus baseline (1s before CS); HRV	HR acceleration for CS+ in accelerators, no difference in decelerators

In a follow-up study, instructed fear acquisition training was employed in a procedurally identical experiment [93]. Here, accelerators showed greater cardiac acceleration to the CS+ during fear acquisition training, while decelerators showed greater deceleration. Both groups, which were identified though cluster analysis, did not show any difference in cardiac responses to the two CSs during extinction.

Furthermore, heart rate acceleration or deceleration in response to the CS+ has been shown to depend on the level of reactivity of one’s aversive motivational system (i.e., the ability to prepare an organism to get away and to motivate avoidance [96]), suggesting a link between acceleration and higher reactivity [92]. Therefore, Lopez et al. [92] classified participants with the psychophysiological reactivity test [97], which consists in the presentation of an unexpected aversive stimulus and the analysis of the resulting cardiac response, before having them undergo the fear conditioning task. By analyzing the resulting Cardiac Defense Response [98] the authors were able to form an accelerators and a decelerators group, also suggesting that cardiac acceleration is related to a higher aversive motivational system reactivity and vice versa. Results revealed that accelerators showed greater cardiac deceleration to the CS+ compared to the CS- during the late phase of the fear acquisition training, while decelerators showed no differences. On the other hand, both accelerators and decelerators showed appropriate explicit contingency learning, highlighting that the aversive motivational system reactivity does not impact on contingency awareness. In conclusion, this study reveals how the presence of fear-induced bradycardia is dependent on the reactivity of the defensive system, while explicit knowledge of CS+/US associations is independent from it.

As a last example of studies examining differences between accelerators and decelerators, Sevenster and colleagues [99] aimed at replicating past findings. Before the analysis, participants were split in two groups, depending on heart rate changes in the last two trials of fear acquisition training by means of a cluster analysis. The results show that cardiac acceleration was stronger to the CS+ compared to the CS- in accelerators, while cardiac responses to the two CSs did not significantly differ in decelerators. Importantly, only HR accelerators showed greater FPS potentiation to the CS+ compared to the CS- while decelerators again showed no differences. Based on these data, the authors concluded that cardiac acceleration is related to adaptive fear learning while cardiac decelerators show impaired FPS conditioning. The authors hypothesize that this may occur because of stronger orienting to the CS+, which results in a lack of appropriate defensive reactions [99]. Nonetheless, it is also possible to hypothesize that participants considered as decelerators might not properly acquire fear conditioning per se, since no heart rate nor FPS modulation was observed in these individuals.

Finally, results from this series of studies are sometimes contradictory and controversial, but in the end a general trend seems to appear: two groups of participants can be defined thanks to cluster analyses or other methods (e.g., psychophysiological reactivity test [97]). This allows to identify differences between these two categories, which mainly suggest that cardiac accelerators show more pronounced fear learning, as expressed by greater physiological differential responding to the CS+ and CS-, while decelerators show an impairment as indexed by lower differentiation of the two types of stimuli. The differences are probably determined by the aversive motivational system reactivity, which is higher in accelerators [92].

### Heart period (HP): a more precise measure of fear-induced cardiac variations

**Fig. 2:**
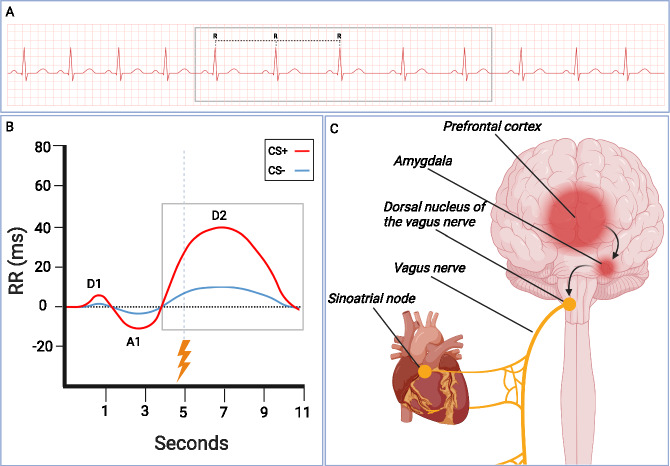
Schematic representation of heart period variations following fear conditioning. **A** Heart period is measured by calculating the distance in milliseconds between consecutive R peaks. **B** Average cardiac responses to CS+ and CS− during fear acquisition training. Starting at the time of CS onset, the classic pattern of early deceleration (D1), acceleration (A1), and late deceleration (D2) can be observed, which persists even after the acquisition training phase, when the CS+ is presented without the US (e.g., during extinction training). The vertical dashed line represents the time of US administration, the horizontal dashed line represents the baseline. **C** When facing a threatening event, a complex interplay between the central and the autonomic nervous systems is set in motion. Under normal circumstances, environmental cues are identified by the prefrontal cortex (PFC), which inhibits sympathoexcitatory networks. Moreover, PFC control over subcortical structures is crucial for HRV regulation [113]. When facing threat, however, these circuits become disinhibited, which in turn allow the emergence of fear responses [139]. The prefrontal cortex and the amygdala govern parasympathetic functioning by regulating the dorsal nucleus of the vagus nerve, innervating the vagus nerve itself, and ending with the sinoatrial node of the heart, whereby fear-induced bradycardia is engendered. This reflects the connections between the central and peripheral nervous systems that reach the heart. The figure was created using BioRender.com.

In the studies that have been described in the previous paragraphs, as in those carried out during the 1900s, heart rate changes were assessed by measuring beats per minute. However, more recently, the use of the heart period (HP) as a measure of cardiac rhythm is becoming more and more popular. It is measured by calculating the distance in milliseconds from an R peak to the following one (R-R interval) (Fig. 2). Heart period is linearly related to autonomic input [100] and allows the identification of specific accelerative and decelerative components in the seconds following stimulus presentation, as will be described in the following studies (see also Table 4 for details).

**Table 4:** A summary of findings on fear-induced bradycardia in studies that use heart period to assess heart rate variations.

Study	Participants (N)	Fear Conditioning Paradigm	Psychophysiological Measures of Fear	CS		US	Analysis Method	Main Findings
Panitz et al. [102]	22	Partial reinforcement delay conditioning	HR	Visual stimuli (emotional and neutral faces)		Loud tone	IBIs	Bradycardia for CS+
Sperl et al. [74]	32	Partial reinforcement delay conditioning	HR, SCR	Visual stimuli (neutral faces)		Shock pulse and loud tone	IBIs	Stronger bradycardia for CS+ for tones
Castegnetti et al. [31]	99	Partial reinforcement delay and trace conditioning	HRV, SCR, FPS	Screen color, rising or falling sounds, Gabor patches		Shock pulse	IBIs	Bradycardia for CS+. Model with higher sensitivity in distinguishing CS+ and CS- than peak-scoring analysis.
Battaglia et al. [104]	50	Partial reinforcement delay conditioning	HRV, SCR	Visual stimuli	Shock pulse		IBIs	Bradycardia for CS+

Since cortico-cardiac coupling, which is the individual covariation of cortical and cardiac activity [101], is known to be modulated by motivationally significant stimuli, Panitz et al. [102] wanted to study if fear conditioned stimuli could influence coupling and if such changes are dependent on the learning experience. The results show that both during fear acquisition training and recall the two CS+ elicited a greater heart rate deceleration compared to the two CS−, which is predicted by the preceding EEG activity derived from the central electrodes position. This study revealed that both the fear acquisition training and extinction processes in fear conditioning can influence cortico-cardiac coupling.

As it was mentioned in the introduction section above, sufficiently arousing CSs are needed in order to properly engender fear-induced bradycardia. Moreover, as the nature of the US plays an important role as well, Sperl et al. [74] aimed at finding what is the best US in terms of strength of conditioned responses and extinction resistance in fear conditioning experiments, especially those with many trials. Results reveal that white noise bursts are more effective in creating a significantly stronger decelerative component for the CS+ compared to the CS-, suggesting that loud noise bursts are more effective than shocks in instantiating fear-induced bradycardia.

**Fig. 3:**
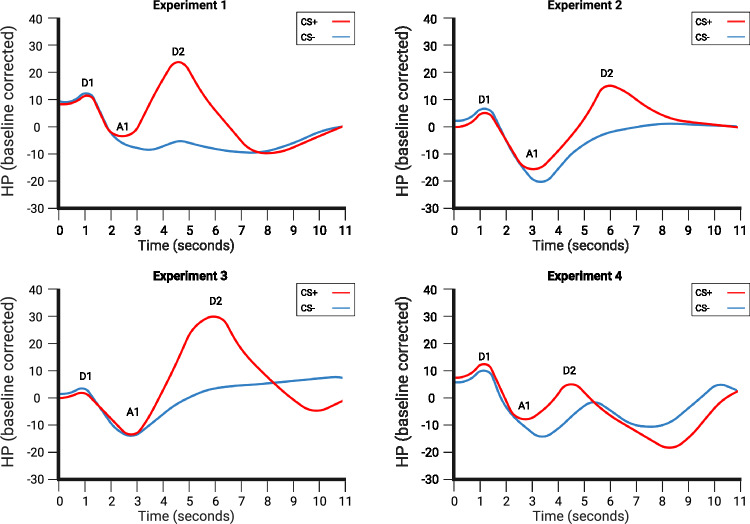
Heart period responses from the experiments by Castegnetti et al. [31]. Each experiment was designed differently with the aim of validating a psychophysiological model that can discriminate between CS+ and CS− based on heart period responses. In fact, in both experiments 1 and 3, heart period responses had a better predicting validity than skin conductance responses, suggesting the feasibility of the model. In each of these graphs, the classic pattern of D1, A, and D2, more pronounced in the case of CS+ presentation compared to CS-, can be observed. This image was adapted from Figure 3 from Castegnetti et al. [31], Psychophysiology. The figure was created using BioRender.com.

The overarching goal of this review is to provide the information necessary to use fear-induced bradycardia as another valuable psychophysiological measure in fear conditioning research. An early example of this idea comes from Castegnetti and colleagues [31], who aimed at creating a computational model for fear-induced bradycardia. To do so, they set three goals: assess if and how heart period responses (HPR) allow to make inferences on fear memories, seek the best way to quantify fear from HPR, and compare the power of SCR and HPR to distinguish between types of CS. The authors first built the methods based on a first delay conditioning experiment, then validated the model with three more experiments. For all the experiments, after exposure to the CS+, participants’ heart rate displayed a classical pattern of a brief early deceleration (D1), followed by a short acceleration (A1), followed by a late and more prominent deceleration (D2) [103] (Fig. 3). On top of that, the D2 component emerges only during CS+ presentation, diverging from the pattern of cardiac response to the CS−. From the data collected in the four experiments authors then proposed a psychophysiological model able to discriminate between CS+ and CS−, in some cases with greater precision than non-model-based methods. More specifically, in experiment 1 and 3, HPR had a better predictive validity than SCR, but the opposite is true for the remaining experiments. The authors explained that this discrepancy could not be due to the different number of trials in the experiments, but rather to the different design choices of the experiments, like the type of CSs used. In experiment 1 and 3, the CSs were different screen colors, while in both experiment 2 and 4, CS were 4 in total, consisting of a simple pair and a complex pair depending on their sensory characteristics. The authors suggested that the alternation of simple and complex stimuli may have influenced the predictive validity of HRP. In summary, this study suggests that psychophysiological models can be a valuable resource in fear-induced bradycardia research, but also warns that the characteristics of the experimental design may influence the outcome. However, the authors suggest that HPR is a robust indicator of fear learning and that model-based approaches complement it well, as the best model they employed outperformed all other model-free approaches in terms of ability to discriminate between CS+ and CS− [31].

**Fig. 4:**
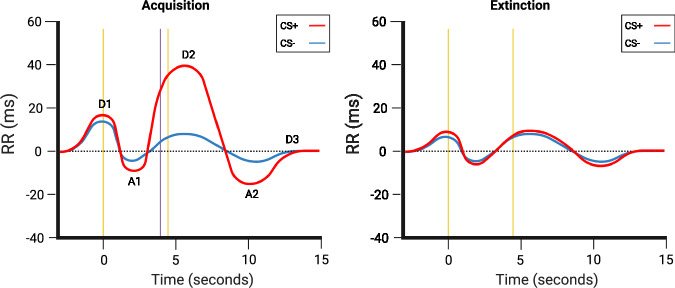
HP responses from Battaglia et al. [104] experiment. Beyond the classical deceleration and acceleration pattern, two new components have been highlighted, a second acceleration (A2) and a third deceleration (D3), bringing HR back to baseline level. The yellow lines represent, from left to right, the time of CS onset and offset, while the purple line represents the time of US administration. As an important note, the onset of the D2 component is concomitant with the time of US administration. This image was adapted from Battaglia et al. [104], Psychophysiology. The figure was created using BioRender.com.

The latest and most recent evidence can be found in a recent work by Battaglia et al. [104], who focused on the spectral components of the cardiac response to assess vagal activity. Since the high frequency (HF) component is linked to the parasympathetic system, and thus provides an index of activity in the vagus nerve, and the low frequency (LF) component emerges from both sympathetic and parasympathetic activity, the analysis of these components holds promise to provide insights into the mechanisms that drive fear conditioned bradycardia. Results revealed the same D1, A1, D2 cardiac response pattern previously described which was greater after viewing a CS+. Furthermore, by expanding the time window of HPR analysis to 15 seconds after CS onset, the authors were able to identify a second acceleration (A2) followed by a third deceleration (D3), bringing the heart period back to its baseline level. The second deceleration, which onset was close to the US presentation, was especially different from the response to the CS−, suggesting how the anticipation of receiving a shock impacts on the heart rate. Moreover, frequency analysis highlighted greater HF changes of power that were significantly larger after CS+ presentation when compared to CS− presentation (Fig. 4). The peak difference could be observed at around the time the shock was delivered to participants. HF components suggest vagal activation, synonymous with heightened sensory intake. Both HPR and frequency differences were only present during fear acquisition training, meaning that extinction was successful in taking those parameters back to a baseline level. These results provide a novel methodology to study HPR changes during fear conditioning by suggesting a direct measure of vagus nerve involvement in response to learned fear.

As studies in this section suggest, the use of HP as a measure for fear-induced bradycardia instead of BPM may provide more generalizable results and better homogeneity between research groups. This is a mandatory feature if fear-induced bradycardia is to be used as an additional measure for the assessment of fear responses, alongside the more renowned and used SCR and FPS. The contributions from each study presented above constitute the foundations for guidelines that will be described in the last section of this review.

### Controversial findings in fear induced bradycardia research

In some cases, fear-conditioned stimuli do not cause a deceleration of heart rate, but rather favor the acceleration or no difference between CS + and CS-. While this could be due to individual differences (e.g., accelerators vs. decelerators), the way heart rate is analyzed determines what is measured and the subsequent results.

For instance, Tröger and colleagues [105], investigated if exposure to a context could alter the conditioning effects involving that context by using Virtual Reality (VR). However, no HR modulation was observed in the fear conditioning paradigm, while both SCR and FPS revealed significant differences between the CS+ and CS-. However, in this study heart rate was calculated as a mean during the different phases of the experiment, instead of being analyzed on a wider time scale following CS onset. Thus, the eventual presence of fear-induced bradycardia could not be assessed.

In another study from the past decade [106] heart rate analysis results showed how prior to fear acquisition training the CS- elicited an accelerated heart rate compared to the CS+, while after fear acquisition training the opposite was true. On the other hand, no other differences were found in the following phases. Here, however, heart rate was measured before and after test phases, instead of being measured throughout the whole experiment. Therefore, the lack of an assessment of post-stimulus heart rate changes prevents from determining the presence of fear-induced bradycardia.

Other contrasting results come from a study [95] which results highlighted that hyperventilators displayed cardiac acceleration during CS+ presentation compared to CS- during the late phase of fear acquisition training, while no difference was observed in hypoventilators. Even though this looks like a contrasting result in comparison to fear-induced bradycardia, as in this study CS+ presentation fostered an accelerated HR, a key detail may be the explanation for the discrepancy. CSs were presented for 8 seconds, and the US was delivered at CS offset. Authors analyzed only the 8 seconds during which the CS was on the screen, but not the time during and after the US presentation. More recent studies [31, 102, 104] analyze heart rate in a time window that goes beyond US offset, which allows to correctly identify all accelerating and decelerating components of the conditioned cardiac response [103]. Therefore, future studies will need to take into account this detail by focusing on a larger trial time window when analyzing HR data.

A last study that did not find evidence supporting fear-induced bradycardia comes from Burger and colleagues [86], which aimed at exploring the effects of tVNS on fear extinction in humans. However, we will not further elaborate since the fear acquisition training procedure in general seems to have been unsuccessful (i.e., failed to produce physiological differences as measured by both FPS potentiation and cardiac activity).

In conclusion, the different results coming from these studies probably depend on differences in the way heart rate responses were analyzed. In order to identify fear-induced bradycardia, besides a large enough analysis time window, it is necessary to record heart rate throughout the whole experiment and use data for the individual heart beats instead of averaging their values. Naturally, as in the case of Burger and colleagues' study [86], an unsuccessful fear acquisition training prevents from moving forward.

## Heart rate variability

Heart rate variability (HRV) is the fluctuation in the time intervals between adjacent heartbeats [107]. It is interpreted as an indicator of prefrontal inhibitory capacity and adaptability to environmental changes, as proposed by the neurovisceral integration model [55, 108, 109]. Resting-state HRV has been shown to be lower in individuals with anxiety disorders [110, 111]. Individuals with low HRV have difficulties to detect safety, an impairment that may derive from an inability to disengage threat detection which perpetuates arousal even in the absence of a real threat [55]. Accordingly, Pappens et al. [112] hypothesized that HRV may predict fear extinction and safety learning success. Fear acquisition training data revealed enhanced FPS response to the CS+ in the paired group, in which the CS+ was immediately followed by the US, but not in the unpaired group, where the CS+ and the US were temporally separated by a long inter stimulus interval. Moreover, in the unpaired group only, participants with high HRV showed a decreasing linear trend in FPS responses to the CS+, which may indicate better safety learning. No effects were present for the paired group. Results from the extinction phase reveal an expected decreasing trend in CS+ FPS responses in the paired high HRV group, which was not present in the paired low HRV group nor in the two unpaired groups. This data suggests that higher HRV is linked to better safety learning, both during fear acquisition training and extinction phases.

The study above suggests differences between high and low HRV participants regarding safety learning. To explore this concept in more detail, Wendt and colleagues [113] designed a study that could assess the participants’ ability to inhibit the responses to previously fear conditioned stimuli when paired with the CS-, as opposed to when they are presented alone. FPS results show that only participants with high HRV display inhibition during the combined presentation of the CS+ and CS-, compared to the CS+ alone. Furthermore, the higher the participant’s HRV, the greater the reduction in FPS potentiation during CS+/CS- presentations. HRV is also linked to extinction learning: only high HRV participants showed extinction of FPS responses. This means that low HRV participants failed to show successful extinction, even if they were told that they would not receive any more shocks during this last phase, and electrodes were removed. These results highlight the relationship between HRV levels and the ability of inhibiting a fear conditioned response.

As both studies described above have demonstrated, lower levels of HRV seem to be linked to deficiencies in the extinction of fear conditioned responses. In both cases, the results were assessed by means of FPS, while SCR responses failed to show effects involving HRV levels, possibly because the subcortical defense system of low HRV individuals is less efficiently inhibited when a threat signals become safe [113]. To replicate and extend these findings, Wendt et al. [114] conducted two follow-up studies. First, the authors aimed at replicating their previous findings by using the same paradigm with instructed and uninstructed extinction training. While no differences in FPS are observed between high and low HRV during uninstructed extinction, during instructed extinction low HRV participants display higher FPS responses to the CS+. These data reveal that low HRV is related to a deficit in the processing and integration of explicit safety information.

Since in both previous studies from Wendt’s group the instructed extinction phase was preceded by the removal of shock electrodes, the second study [114] included a group of participants that underwent extinction training while still being connected to the electrodes, representing the possibility of still receiving shocks. Results reveal, like the first study, an association between HRV and CS potentiation only during instructed extinction. During the early phase of instructed extinction, low HRV individuals display higher FPS responses to the CS- compared to high HRV individuals, and during the late phase they show higher FPS responses to the intertrial interval as well, suggesting higher disposition to defensive responding. It is important to note, however, that both instructed extinction groups (i.e., participants that had shock electrodes removed and were instructed that no further shocks would be administered and participants which received the same instructions but were still connected to the stimulator) were pooled in a single “instructed extinction group”, therefore suggesting no differences between the two groups. The authors suggest that this data corroborate the idea that the association between HRV and CS potentiation reflects the ability to integrate cognitive information, thus additional information is not helpful in fostering safety.

The studies above have shown the relationship between HRV and safety learning, suggesting that low HRV is linked with deficient safety learning. However, no insight was given regarding the contributions of the autonomic nervous system to this component and its relationship with fear-induced bradycardia. The study by Battaglia et al. [104] that was described in the previous paragraphs helps shedding light in this regard, by measuring vagal activity directly by means of spectral analysis and machine learning algorithms. Spectral analysis was used to distinguish between the frequency specific contributions of the sympathetic and parasympathetic system on HRV. This was achieved by using a point-process modeling algorithm [115] which computes instantaneous estimates of HRV. Moreover, it allows to compute the distribution of spectral powers [116]. Consequently, it is possible to determine the intensity of high frequency (HF) components, which provide a direct index of vagal activity, and low frequency (LF) components. Results show how rapid and transitory vagal activity, as measured by a cluster of power contribution in the HF band at around the time of US administration, determines the emergence of fear-induced bradycardia. Data from this study suggests that even though HRV is under the control of both the sympathetic and parasympathetic nervous system [100, 117], it is the parasympathetic contribution that engenders fear-induced bradycardia, reflecting better sensory intake and preparedness to negative outcomes. Moreover, it not only suggests that the vagus nerve has a crucial role in fear conditioning [56, 89, 118], but also that it is possible to systematically investigate and quantify its selective involvement in human fear conditioning.

The results from these studies reveal HRV as an important component that influences the effectiveness of safety learning. The neurovisceral integration model [55] suggests that HRV is a marker of the functionality of a system that integrates physiological, affective, and cognitive processes for appropriate responses to the environment. Therefore, a lower HRV level may be associated with a lower ability of integrating cognitive information coming from verbal instructions with physiological processes, which is further supported by the finding that removing shock electrodes does not modify the association between HRV and CS potentiation [114]. Moreover, even though both the sympathetic and parasympathetic nervous systems contribute to HRV, only the latter is involved in fostering fear-induced bradycardia, as highlighted by spectral analyses [104]. Taken together, these findings suggest HRV is an important component involved in modulating safety learning.

## Discussion

Psychophysiological indices are of the utmost importance in fear conditioning research, as they have the great advantage of not being biased by self-report [12, 18]. The most popular indices used are the skin conductance response (SCR) and the fear potentiated startle (FPS), while heart rate (HR), despite being known for centuries, has rarely been employed as an index of fear conditioned responses. However, recent advances have led to the use of fear induced bradycardia as a valid additional methodology to study fear learning. Importantly, HR has also been associated with freezing-like behavior in humans, as both fear-induced bradycardia and freezing are engendered by greater parasympathetic involvement [80].

The history of fear induced bradycardia dates back a century, and rapidly evolved especially in the last 20 years to become an interesting measure of fear in fear conditioning experiments. From being a secondary measure, used alongside SCR and FPS, it has evolved to the point where it can be viewed as a standalone methodology to study the dynamics of human fear conditioning. The most recent studies have shown the potential of HR change measures in fear conditioning research, and this review aimed at summarizing this work, by presenting evidence in a concise manner and highlighting the common features of these endeavors. Thanks to these advances, it is possible to define fear-induced bradycardia as a unique measure to be ranked among the more popular alternatives. Notably, it is viable in experiments with a high number of trials [74], suggesting it is not subject to habituation like SCR [12] and allows to determine the specific contributions of the autonomic nervous system [104]. An important next step is to focus on the reliability of this measure – also in relation to more established measures. Recent studies, however, have highlighted some unique strengths of HR measures.

First and foremost, a key feature that differentiates fear-induced bradycardia from other methodologies is its ability to directly index specific vagal activity, therefore suggesting that fear induced bradycardia is mediated by the parasympathetic system [104]. This finding is further corroborated by the idea that transcranial vagus nerve stimulation (tVNS) enhances fear extinction as measured by a faster attenuation of cardiac deceleration [84].

Second, a series of studies from different researchers suggested that participants can be categorized in accelerators or decelerators by using cluster analyses [75, 92–95]. Results from these studies are in part contradictory, but cardiac accelerators seem to show better fear learning while decelerators show more difficulties in distinguishing the CS+ from the CS-. It is important to note, however, that in these studies the analysis was focused on HR changes happening only during CS presentation. On the other hand, the more recent studies analyzed here focused on bigger time windows, extending past CS offset [31, 102, 104]. As the D2 component generally arises around the time of US administration, by focusing on a restricted time window it could be cut out. This may have thus led to the measurement of only the D1 and A1 components, therefore leaving out the D2, the most prominent deceleration, from the analysis, making comparisons with other studies problematic. Anyway, future studies may help in clarifying the differences between accelerators and decelerators, for example by using HP to study HR variations, which will lead to more accurate measurements thanks to a faster sampling rate and the identification of the aforementioned accelerative and decelerative components, and by including a bigger trial time window in their analysis, so that HR modulations happening after CS offset are taken into account as well, which are present up to 15 seconds after CS presentation [104].

As a matter of fact, during recent years the use of heart period (HP) instead of beats per minute (BPM) to measure HR changes has become more popular. It is a measure of the distance in milliseconds from an R peak to the other and is related to autonomic input [100], allowing the identification of specific accelerative and decelerative components. Newer evidence has shown that using HP as a measure for fear induced bradycardia leads to more precise data, as HP allows to employ a faster sampling rate [119] compared to BPM measurements, which require large time bins, and greater convergence in results between different research groups [31, 74, 102, 104, 120].

Lastly, another useful index in fear conditioning experiments can be heart rate variability (HRV), a component that is associated with a lower ability of integrating information from verbal instructions with physiological processes [114]. Accordingly, elevated defensive responses in safety conditions are a reflection of a generalized feeling of unsafety, which is a core component of chronic stress and reliant on internal bodily processes instead of external stimuli [121]. Thus, low HRV participants may show deficient safety learning due to a predominant internal physiological activation that cannot be overwritten by external cues [114].

### Open questions

A pressing question remains unanswered: does respiration affect fear-induced bradycardia? It is known that breathing can influence HRV [122, 123]. One can hypothesize that the response to the CS+ can impact on HRV because of two possibilities: either directly, because of parasympathetic control of heart rate signal, or because of an indirect effect of breathing on heartbeat variations [104]. However, it is difficult to say for certain if fear-induced bradycardia is influenced by breathing, as no studies specifically investigated this issue. Nonetheless, future endeavors focusing on this issue by controlling for breathing may help clarify this point.

To our knowledge, no study concerning the reliability of HP measurements to assess fear-induced bradycardia has been carried out to this day, which constitutes a major limitation. In the future, investigations on this issue will help in furthering the knowledge regarding fear-induced bradycardia and its study.

## Guidelines

Classically, the most renowned guidelines for heart rate studies in humans have been proposed back in 1981, in a work by Jennings and colleagues [124]. Besides their historical importance, these guidelines still constitute a valuable resource. However, here we aim to highlight a series of recommendations and precautions researchers should consider when designing experiments in humans that use heart rate as the primary dependent variable. Remarkably, many of their recommendations are based on solid pieces of advice that must be followed to ensure proper experimental designs (for instance, suggestions regarding participant selection and exclusion criteria, setting and stimuli considerations, and recording and analysis parameters). Notwithstanding, with our review, we want to update and complement these recommendations by providing guidelines and further advice specifically focused on fear conditioning experimental designs. To do so, we distilled the evidence from the more recent studies that were analyzed in the present manuscript.

- The use of heart period (HP) instead of beats per minute (BPM) is recommended, as it is more precise. While BPM change has to be measured in time bins [78, 84], HP allows for more temporally precise measurements [31, 104]. Moreover, the use of spectral analyses is highly encouraged, as it allows to distinguish between the contributions of the parasympathetic and sympathetic systems on HRV.
- It is important that the trial time-window for the analysis includes the US, as fear-induced bradycardia onset is related to US presentation [31, 104]. This will ensure that the study will capture all decelerative and accelerative components, which could be left out otherwise.
- It is suggested to employ within subjects designs to enhance statistical power, as heart rate variations are subject to individual differences [125].
- In our opinion, a 5-second trial including US is to be considered optimal, even with co-recording of SCR or FPS. In the case of FPS registration, we suggest including some un-probed trials, in order to have a set of trials where cardiac activity is not influenced by the presentation of the acoustic sound (e.g., Szeska et al., [84]).
- We recommend using neutral stimuli, as threatening stimuli may influence a participant’s physiological activation based on their predisposition towards what is represented on screen. Geometrical shapes [78], Gabor patches [31], and neutral faces [74] are all appropriate choices.
- It is necessary to employ a sufficiently threatening US, carefully weighed on the participant’s individual pain threshold. Both shocks to the wrist and loud noises are effective, but one may prefer using the latter in case of an experiment where a high number of trials is required, for example for protocols that require co-recording of neurophysiological methods like electroencephalography or magnetoencephalography in order to ensure an adequate signal to noise ratio [74].
- We recommend having more CSs presentations than in more classical SCR or FPS studies, as it seems that fear induced bradycardia needs more time compared to other psychophysiological indices to occur [104]. 40 are enough to promote fear induced bradycardia [102, 104]. It is important to note, however, that the amplitude of the SCR strongly decreases over many presentations of the same cues (i.e., habituation; Boucsein et al., [126]; Dawson et al., [127]). Therefore, this needs to be considered for future research, and it will need to be taken into account when designing paradigms that involve recording of both HR and SCR.
- Model-based estimates provide an additional method to homogenize heart response scoring through different studies and better identify commonalities between them. Castegnetti and colleagues’ work [31] provides an in-depth look at the matter. However, as we have not tested the efficacy of model-based approaches over other methods, we encourage future research to investigate this issue.
- Data sharing and a common data format would be beneficial to guarantee transparency, reproducibility, and cooperation between research groups [128] and facilitate cumulative knowledge generation.

## Conclusion

In conclusion, in this review we summarized evidence for the role of fear-induced bradycardia in human fear conditioning experiments across a wide range of paradigms and methodologies. Our goal was to encapsulate all relevant studies to help to better understand this phenomenon by drawing theoretical and methodological conclusions. We discussed all relevant studies thus far in coherent sections to promote clarity about how each work contributed to the development of this multi-domain field. Moreover, we provided insights about some analysis precautions to consider when investigating fear-induced bradycardia, which will be useful in future research. Finally, we suggested specific guidelines to foster greater homogeneity and ease of interpretation of results from different research groups. Our objective in sharing these insights is to help drive future research in this field, which in turn will ensure greater depth of understanding of this phenomenon and provide the basis to translate its use to the clinical field, as both fear-induced bradycardia and HRV hold potential to serve as biomarkers for the study and treatment of various human psychiatric disorders characterized by aberrant fear conditioning, such as depression [129], anxiety [130], specific phobias [131], panic disorder [132], and post-traumatic stress disorder [133]. What about future perspectives? What are the gaps in the scientific literature that future work should address most urgently? As it stands, neuroscientific investigations into the reliability and validity of HR responses to assess fear-induced bradycardia are needed, as well as more, high quality data collected from new fear conditioning studies.

Notably, HR responses have shown to provide insights where other measures failed to detect change. For instance, while SCR showed no evidence for differences between conditioned stimuli in psychiatric populations [134] or between healthy and socially anxious participants [135], HR measures highlighted the presence of such differences. Hence, it could be hypothesized that the mechanisms that govern heart rate regulation in response to aversive stimuli are partially different from those of other common psychophysiological measures. Thus, a better understanding and wide use of fear induced bradycardia as a psychophysiological measure may help in disentangling these underlying mechanisms. Moreover, fear induced bradycardia is present even after an elevated number of trials [31, 74]. Other measures, like SCR, are instead subject to decline over time because of habituation [126, 127]. As such, it can be useful when applied to paradigms that require many trials. Furthermore, analyses on the spectral components of heart rate can provide insights into the specific contributions of the autonomic nervous system, allowing to study how its influence can impact fear related responses [104].

Future investigations will have a two-sided impact on the future of HR responses evaluation. On one end, they will lead to the development of a common and solid ground for scientists of different fields when studying conditioned responses by means of fear-induced bradycardia, ensuring more homogeneity. These insights will greatly help to shape the future of research in this field, providing valuable knowledge for novel scientific endeavors. On the other end, new findings will also be of remarkable importance in the clinical setting. As it stands, genetic differences [136] as well as psychiatric disorders [137, 138] impact the way fear-induced bradycardia is manifested. Consequently, a better understanding of this phenomenon could provide with a reasonable, additional instrument in the investigation of psychiatric illnesses, with a specific focus on the diagnostic process, rehabilitation treatments and outcome assessments.
